# 重组人血管内皮抑制素在晚期肺鳞癌治疗中的临床应用

**DOI:** 10.3779/j.issn.1009-3419.2016.10.06

**Published:** 2016-10-20

**Authors:** 镨元 邢, 学志 郝, 兴胜 胡, 燕 王, 峻岭 李

**Affiliations:** 100021 北京，国家癌症中心/中国医学科学院北京协和医学院肿瘤医院 National Cancer Center/Cancer Hospital, Chinese Academy of Medical Sciences and Peking Union Medical College, Beijing 100021, China

**Keywords:** 肺肿瘤, 鳞状细胞癌, 抗血管生成药物, 重组人血管内皮抑制素, 化疗, Lung neoplasms, Squamous cell carcinoma, Anti-angiogenesis agents, Recombinant human endostatin, Chemotherapy

## Abstract

**背景与目的:**

肺鳞癌是非小细胞肺癌常见的病理类型，晚期肺鳞癌是一种无法治愈的恶性肿瘤。抗血管生成药物与传统化疗联合能够为患者带来生存改善。本研究分析了重组人血管内皮抑制素（恩度）联合化疗治疗晚期肺鳞癌的疗效及安全性。

**方法:**

回顾性分析中国医学科学院肿瘤医院内科2011年11月-2015年5月采用人血管内皮抑制素联合传统化疗方案治疗的15例晚期肺鳞癌患者的近期疗效、毒副反应及无进展生存时间。

**结果:**

14例可评估患者中疗效评价最佳即为部分缓解5例（35.7%）、疾病稳定7例（50.0%）、疾病进展2例（14.3%），客观缓解率为35.7%，疾病控制率为85.7%，中位无进展生存为9.3个月。全组患者治疗耐受良好，3度不良反应表现为中性粒细胞减少（2/15, 13.3%）和呕吐（1/15, 6.7%），其余不良事件均为1度/2度。

**结论:**

重组人血管内皮抑制素联合化疗治疗晚期肺鳞癌可取得较好的客观疗效并且安全性良好。

## Abstract (en)

**Background and objective:**

Squamous cell carcinoma (SCC) is a common pathological type of nonsmall cell lung cancer, and advanced lung SCC is incurable. Chemotherapy combined with anti-angiogenesis agents can prolong the patients' survival time. Te aim of the study was to analyze the efcacy and safety of recombinant human endostatin (Endostar) in treating advanced lung SCC.

**Methods:**

We retrospectively analyzed the short-term efficacy and toxicity of recombinant human endostatin combined with traditional chemotherapy regimens in treating 15 advanced lung squamous cell carcinoma patients in Department of Medical Oncology retrospectively, Cancer Hospital, Chinese Academy of Medical Sciences from November 2011 to May 2015. Treatment-related survival was also analyzed.

**Results:**

Among the evaluble 14 patients, the best overall response was partial response in 5 patients (35.7%), stable disease in 7 patients (50.0%), and progressive disease in 2 patients (14.3%). Te objective response rate (ORR) was 35.7%, and disease control rate (DCR) was 85.7%. Te median progression-free survival (PFS) was 9.3 months. Te main grade 3 toxicity was neutropenia (2/15, 13.3%) and vomiting (1/15, 6.7%).

**Conclusion:**

Chemotherapy combined with recombinant human endostatin enabled good objective response in advanced SCC patients and had well security.

肺癌的年发病人数在全球范围呈现逐年上升的趋势，最新流行病学数据显示无性别差异，男性、女性肺癌发病率及死亡率均居于恶性肿瘤首位^[1]^。肺癌中近85%为非小细胞肺癌（non-small cell lung cancer, NSCLC），其中鳞状细胞癌（squamous cell carcinoma, SCC）占30%左右^[2]^。随着越来越多肿瘤驱动基因的发现，表皮生长因子受体酪氨酸激酶抑制剂（epidermal growth factor receptor tyrosine kinase inhibitor, EGFR-TKI）、间变淋巴瘤激酶（anaplastic lymphoma kinase, ALK）抑制剂为代表的靶向治疗药物为肺癌患者带来生存时间的延长。但是，由于肺鳞癌较少发生上述驱动基因状态改变，因此较非鳞NSCLC的治疗药物选择少，生存期无大幅度改善^[3, 4]^。目前，国内获得抗肿瘤治疗适应症的靶向药物中抗血管生成药物是晚期肺SCC除传统细胞毒药物外的新的治疗手段。

重组人血管内皮抑制素（恩度）为血管生成抑制剂类生物制品，1999年由我国自主研发并于2005年被国家食品药品监督管理局批准联合化疗应用于复发和转移NSCLC的治疗。重组人血管内皮抑制素通过作用于血管内皮细胞，以抑制形成血管的内皮细胞的迁移，抑制肿瘤新生血管的生成，阻断肿瘤细胞的营养供给，从而达到抑制肿瘤增殖或转移^[5]^。2013年，学者Meng等^[6]^通过检测经重组人血管内皮抑制素治疗后的Lewis肺癌小鼠模型中肿瘤的细胞凋亡、微血管密度、壁细胞血管形态以及肿瘤缺氧的情况，提出重组人血管内皮抑制素可能通过降低微血管密度及增加血管内皮周细胞覆盖从而对肿瘤血管进行重塑，继而出现血管“正常化”现象。这种抗血管治疗后早期肿瘤血管短期的“正常化”现象，使得发育不成熟、低灌注的肿瘤新生血管短期血流量升高，进一步提高对药物以及氧的运输能力，使得化放疗联合重组人血管内皮抑制素治疗后疗效获得明显提高^[7]^。本研究旨在进一步探讨重组人血管内皮抑制素联合传统化疗治疗晚期肺SCC的疗效及安全性优势。

## 1 材料与方法

### 1.1 临床资料

收集2011年11月-2015年5月中国医学科学院肿瘤医院内科接受完整重组人血管内皮抑制素联合化疗的晚期肺SCC患者资料15例进行分析。全部患者临床病理资料完整，经病理科确诊为肺鳞状细胞癌（无其他病理混合成分），在我院完成全部治疗计划，按计划定期随访安全性数据及影像学评估，直至疾病进展。15例患者中男性14例，女性1例；年龄范围40岁-71岁，中位年龄59岁；全部患者美国东部肿瘤协作组（Eastern Cooperative Oncology Group, ECOG）0分-1分；吸烟史10例，不吸烟5例；治疗前临床分期为Ⅲb期2例，Ⅳ期13例；初诊合并咯血或痰中带血2例；合并高血压病3例；糖尿病2例；14例患者为一线接受重组人血管内皮抑制素联合化疗的治疗，1例患者为二线治疗。主要转移部位包括纵隔淋巴结（10/15, 66.7%）、肺（6/15, 40.0%）、骨（5/15, 33.3%）、胸膜（3/15, 20.0%）、肾脏（2/15, 13.3%）、肝脏（1/15, 6.7%）、脑转移（1/15, 6.7%）、远处淋巴结（2/15, 13.3%）。化疗采用GP方案（吉西他滨联合顺铂）8例（53.3%）、紫杉醇联合顺铂方案3例（20.0%）、多西他赛联合顺铂2例（13.3%）、多西他赛单药1例（6.7%）、EP方案（依托泊苷联合顺铂）1例（6.7%）。接受同步放化疗序贯巩固化疗模式治疗的2例（13.3%），1例为EP方案同步放疗，1例为紫杉醇联合顺铂方案同步放疗（表 1）。

**1:** 患者基线特征 Baseline demographic and disease characteristics

Characteristics	Recombinant human endostatin+chemotherapy (*n*=15)
Age in years, median (range)	59 (40-71)
Male/female, *n* (%)	14 (93.3)/1 (6.7)
ECOG PS 0/1, *n* (%)	8 (53.3)/7 (46.7)
Smoking/non-smoking, *n* (%)	10 (66.7)/5 (33.3)
Metastatic sites, *n* (%) Mediastinal lymph nodes/lung/bone/renal/liver/CNS/other lymph nodes	10 (66.7)/5 (40.0)/5 (33.3)/3 (20.0)/2 (13.3)/1 (6.7)/1 (6.7)/2 (13.3)
Chemotherapy agents, *n* (%) Gemcitabine Docetaxel Paclitaxel Cisplatin Other	8（53.3） 3（20.0） 3（20.0） 14（93.3） 1（6.7）
ECOG: Eastern Cooperative Oncology Group; PS: performance score; CNS: central nervous system.	

### 1.2 方法

#### 1.2.1 治疗方案

重组人血管内皮抑制素采用静脉输液，7.5 mg/m^2^/次，1次/日，连续用药14天，休息7天的给药方式，与化疗药物联合使用。化疗方案均为标准的3周方案，吉西他滨1, 000 mg/m^2^，第1、8天，静脉滴注；紫杉醇175 mg/m^2^，第1天，静脉滴注；多西他赛75 mg/m^2^，第1天，静脉滴注；顺铂75 mg/m^2^，分为2天-3天静脉滴注；依托泊苷120 mg/m^2^，第1-3天，静脉滴注。每2个治疗周期接受1次影像学（电子计算机断层扫描/磁共振成像）评估。治疗周期中患者定期进行安全性访视，按计划进行肿瘤评估直至出现疾病进展。

#### 1.2.2 疗效及不良反应评价

疗效的客观判断标准按照实体瘤的评价标准（Response Evaluation Criteria in Solid Tumors, RECIST 1.0）进行评定，观察指标包括完全缓解（complete response, CR）、部分缓解（partial response, PR）、稳定（stable disease, SD）和进展（progressive disease, PD），客观有效率（objective response rate, ORR）和疾病控制率（disease control rate, DCR）。无疾病进展生存时间（progression-free survival, PFS）定义为从首次给药至有客观证据证实的疾病进展的时间。不良反应根据2003年美国国立癌症研究所通用不良事件术语标准（National Cancer Institute-Common Toxicity Criteria for Adverse Events, NCI-CTCAE）3.0版进行评估。

### 1.3 统计学方法

采用SAS 9.2统计分析软件进行数据处理。定量指标的描述采用均数或中位数。分类指标的描述采用例数及百分数。

## 2 结果

### 2.1 近期疗效及生存结果

全组15例晚期肺SCC患者接受重组人血管内皮抑制素治疗的周期数为1周期-6周期，中位治疗4周期。接受化疗的周期数为1周期-29周期，中位化疗周期为4周期。1例患者完成第1治疗周期后，由于出现3度恶心/呕吐不良反应放弃继续治疗，仅记录安全性数据，其余14例患者可进行疗效评估。PR为5例（35.7%），SD为7例（50.0%），PD为2例（14.3%），ORR为35.7%，DCR为85.7%。随访时间截至2016年7月1日，中位随访时间为13.2个月（3个月-56.5个月），中位PFS为9.3个月，中位OS为13.2个月。

### 2.2 不良反应

本组患者采用重组人血管内皮抑制素联合化疗的主要不良反应表现为：中性粒细胞计数减低、恶心/呕吐、食欲减退、皮疹、乏力，无明确心脏毒性发生，未增加出血事件的发生，3度不良反应少见，无4度不良反应发生，多为1度/2度不良反应（表 2）。

**2:** 常见不良反应发生率 Incidence of the common treatment-related adverse events

Adverse event	Total grades, *n* (%)	Grade 3/4, *n* (%)
Neutropenia	4 (26.7)	2 (13.3)
Nausea/vomitting	6 (40.0)	1 (6.7)
Fatigue/asthenia	3 (20.0)	0 (0)
Anorexia	5 (33.3)	0 (0)
Rash	1 (6.7)	0 (0)

## 3 讨论

在恶性肿瘤生长与转移中，新生肿瘤血管形成发挥着至关重要的作用，早在1971年Folkman教授^[8]^即提出恶性肿瘤的生长与转移依赖于新生血管生成这一学说。贝伐珠单抗（Bevacizumab）是一种血管内皮细胞生长因子（vascular endothelial growth factor, VEGF）抑制剂，已在国内获批晚期NSCLC适应症，但由于致命性肺出血的发生而禁用于肺SCC^[9, 10]^。在一项Ⅱ期临床研究AVF0757g中，有9例患者死于治疗相关性毒性，主要原因为大咯血、肺出血、肝衰竭等^[10]^。AVF0757g研究中贝伐珠单抗治疗组有6例发生咯血，其中4例为致命性，并发现这种致命性肺出血不良反应的发生与SCC组织间的关联性，因此在后期的贝伐珠单抗相关研究中排除肺SCC患者。索拉菲尼（Sorafenib）是一种口服的小分子多种激酶抑制剂，同时作用于肿瘤细胞及肿瘤脉管系统而发挥抗肿瘤作用。索拉菲尼抑制在增生的血管内皮细胞生长因子受体2、淋巴内皮细胞生长因子受体3和平滑肌细胞血小板衍生生长因子受体（platelet-derived growth factor receptor, PDGFR）β中表达的靶受体的磷酸化，这些细胞是新生血管的组成成分。VEGF和PDGFR是肿瘤新生血管生成所必需的^[11, 12]^。索拉菲尼在晚期NSCLC治疗中进行了多项临床试验探索（ESCAPE研究、REAP研究、NEXUS研究、MISSION研究），总体来说SCC组各级不良反应的发生率均高于安慰剂组，且高于非SCC组，其中一项Ⅲ期随机研究ESCAPE^[13]^中期分析发现索拉菲尼治疗组SCC患者的治疗相关死亡风险增加（HR=1.85）。雷莫如单抗（Ramucirumab）是VEGF受体2抑制剂，二线治疗中标准化疗多西他赛联合雷莫如单抗后较单药多西他赛能提高晚期NSCLC中位总生存时间（10.5个月*vs*9.1个月，HR=0.86，95%CI: 0.75-0.98，*P*=0.023）及中位无进展生存时间（4.5个月*vs*3个月，*P*＜0.000, 1），据此雷莫如单抗获得晚期NSCLC二线治疗适应症。这项Ⅲ期临床研究（REVEL）^[14]^中入组晚期肺SCC患者比例为25%左右，但在肺SCC亚组分析中这种生存优势并不明显（9.5个月*vs*8.2个月，HR=0.88，95%CI: 0.69-1.13）。后续雷莫如单抗联合紫杉醇、卡铂作为Ⅲb期/Ⅳ期NSCLC一线治疗的Ⅱ期临床研究中，入组了更少的SCC患者（15%左右），并且针对这部分患者的亚组结果至今无公开数据发表^[15]^。由此可见，即使不考虑发生率较高的高血压、蛋白尿等不良反应及高额的治疗费用，贝伐珠单抗和雷莫如单抗这两种通过抑制VEGF/VEGFR达到抗肿瘤血管生成的单克隆抗体药物在晚期肺SCC治疗中的疗效表现亦不尽如人意。

综上可见，绝大多数已经上市的抗血管生成药物因为毒性原因在晚期肺SCC的临床试验中相继惨遭失败。同时，晚期肺SCC已知驱动基因突变率较低，不常规推荐进行*EGFR*突变和*ALK*基因重排检测，无较好的靶向治疗手段，生存率较腺癌低。虽然肿瘤学家们始终坚持不懈的寻找晚期肺SCC相关的高频变异驱动基因及研发相应靶向药物，但是临床实际情况是晚期SCC患者仍然无更好的药物新选择。

重组人血管内皮抑制素的临床前研究^[16]^显示，该药能抑制血管内皮细胞增殖、血管生成和肿瘤生长，还可以直接诱导肺癌细胞凋亡。虽然上述具体机制尚不十分清楚，但随后进行的Ⅰ期-Ⅳ期临床研究中重组人血管内皮抑制素与化疗的联合治疗显示出良好的抗肿瘤疗效及很好的安全性。其中最值得关注的就是对于晚期肺SCC患者，这种抗血管新生药物的使用并未明显增加治疗相关性不良反应的发生，同时提高了ORR、延长了疾病进展时间（time to progression, TTP）。在不良反应方面，重组人血管内皮抑制素常见的药物不良反应（发生率1%-10%）主要有心脏毒性，少见的药物不良反应（发生率＞0.1%而＜1%）主要有消化系统反应、皮肤及附件的过敏反应，未观察到与药物不良反应相关的死亡病例^[5]^。心脏毒性是重组人血管内皮抑制素的剂量限制性毒性的主要表现形式，其中以心律失常最为常见，主要表现为窦性心动过速、房性或室性早搏、房室传导阻滞以及ST-T改变，多见于既往患有冠心病、高血压等基础心血管疾病的患者，停药或对症治疗后均可恢复正常^[16]^。心脏毒性在用药初期少数患者可出现轻度疲乏、胸闷、心慌，绝大多数不良反应经对症处理后可以好转，不影响继续用药，极个别病例因上述症状持续存在而停止用药。本研究的15例患者总体耐受良好，发生的不良反应主要为所采用的化疗方案常见的毒性，且重组人血管内皮抑制素的使用并未增加毒性的发生率及严重程度，未观察到有明确心脏毒性的发生。重组人血管内皮抑制素与化疗联合的疗效评估方面，Ⅱ期研究显示重组人血管内皮抑制素联合化疗NP方案（长春瑞滨联合顺铂）ORR为22.2%-37.0%，中位TTP为151天-166天，与单纯NP方案组比较ORR及TTP均明显延长（0-24.4%，66天-100天）、常见的3度/4度不良反应（毒副反应）发生率差异无统计学意义（*P*＞0.05）^[17, 18]^。随机、双盲、安慰剂对照的多中心Ⅲ期临床研究的亚组分析提示：重组人血管内皮抑制素联合NP方案组共入组晚期肺SCC患者129例（占联合治疗组入组总人数的40.1%），ORR优于单纯NP方案化疗组（37.98% *vs*18.18%, *P*=0.008, 6），TTP延长（6.45个月*vs*3.45个月）^[19, 20]^。重组人血管内皮抑制素上市后的Ⅳ期临床研究入组了2, 725例晚期NSCLC患者，其中SCC患者841例（30.95%）。重组人血管内皮抑制素联合化疗一线治疗晚期肺SCC的ORR为36.0%，临床获益率（clinical benefit rate, CBR）为83.3%，中位TTP为8.16个月，中位生存时间为16.89个月。全部肺SCC患者均保持良好的耐受性，未发生重度咯血，1度-2度咯血发生率仅为2.73%。后续很多学者针对重组人血管内皮抑制素与不同化疗方案，如GP方案、TC方案（紫杉醇联合卡铂）、含培美曲塞方案等的联合，以及在晚期NSCLC治疗不同阶段（一线、二线、三线及以上）使用重组人血管内皮抑制素的疗效及安全性进行了进一步深入探索^[21-23]^。本研究中可评估疗效的患者ORR为35.7%，DCR为85.7%，中位PFS为9.3个月，中位OS为13.2个月，与既往研究结果相符合。

目前获批治疗NSCLC适应症的抗血管生成药物中，针对肺SCC患者而言重组人血管内皮抑制素无疑是疗效确切，安全性最好的药物。遗憾的是，迄今无一项前瞻性随机临床研究是针对晚期肺SCC患者使用抗血管生成药物进行系统性的安全性及疗效验证。而本研究恰恰重点关注了晚期肺SCC患者临床应用重组人血管内皮抑制素与化疗联合的有效性及安全性，初步得到重组人血管内皮抑制素这种抗血管生存药物在SCC治疗中安全有效的结论，为后续临床广泛应用及开展前瞻性、大样本、随机临床研究提供重要依据。
